# EGFR mutation analysis on circulating free DNA in NSCLC: a single-center experience

**DOI:** 10.1007/s00432-021-03658-8

**Published:** 2021-05-18

**Authors:** Anna Ianza, A. Di Chicco, C. Biagi, F. Giudici, A. Dicorato, A. Guglielmi, F. Variola, S. Tomasi, G. Roviello, D. Generali, F. Zanconati

**Affiliations:** 1 Department of Medical, Surgery and Health Sciences, University of Trieste, Piazza Ospitale 1, 34129 Trieste, Italy; 2 Azienda Sanitaria Universitaria Goriziano Isontina, Cattinara Academic Hospital, University of Trieste, Strada di Fiume 447, 34149 Trieste, Italy; 3 Breast Cancer Unit and Translational Research Unit, ASST Cremona, Cremona, Italy

**Keywords:** EGFR, Liquid biopsy, cfDNA, Target therapy, Lung cancer

## Abstract

**Purpose:**

Monitoring mutation status in circulating free DNA (cfDNA) during target therapy could hold significant clinical importance in non-small cell lung cancer (NSCLC). Our aim is to establish if EGFR mutational status change on cfDNA has predictive value that can impact clinical management of NSCLC patients care.

**Methods:**

This study included 30 patients with EGFR-mutated NSCLC. Blood samples were collected at diagnosis (T0) and in 19 patients during therapy (T1).

**Results:**

Concordance between T0 and T1 EGFR mutation status for patients evaluable for both samples (*n* = 19) was 79%, with a sensitivity of 100% (95% CI: 55.5–100.0) and specificity of 60.0% (95% CI: 26.2–86.8). For the patients in oncological therapy with targeted drug and with T1 sample available (*n* = 18), survival outcomes were evaluated. For both mutation-negative T0 and T1 patients, 12-month progression-free survival (PFS) was 66.7% (95% CI: 27.2–100.0) and 12-month overall survival (OS) was 100% (95% CI: 1.00–1.00); for patients mutated both at T0 and T1, PFS was 22.2% (95% CI: 6.5–75.4%) and OS was 55.6% (95% CI: 20.4–96.1%).

**Conclusion:**

EGFR mutation status can be assessed using cfDNA for routine purposes and longitudinal assessment of plasma mutation is an easy approach to monitor the therapeutic response or resistance onset.

## Introduction

Most lung cancers (85%) are classified as Non-Small Cell Lung Cancer (NSCLC) and most NSCLC patients are at an advanced stage when diagnosed (Testa et al. 2018). The study of the molecular characteristics of lung tumors has highlighted a specific role of some genes that represent important therapeutic targets, including EGFR (Epidermal Growth Factor Receptor) (Rodgers 2014). In NSCLC (in particular in 10–15% of adenocarcinomas of Caucasian patients and in 40% of Asian patients), activating EGFR mutations have been identified for exons 18, 19, 20 and 21, the presence of which represents the most important predictive factor for the adoption of molecular target therapies with specific EGFR tyrosine kinase inhibitors (EGFR-TKI) (Marchetti et al. 2015). On the other hand, the point mutation in exon 20 (T790M) indicates resistance to EGFR-TKIs and poor prognosis (Wang et al. 2016).

Surgery is the elective treatment in the early stages of disease; resections are indicated only with curative intent, where tumor removal can be achieved with a surrounding healthy margin of tissue, histologically confirmed. Since most NSCLC patients are diagnosed at an advanced stage, surgery is no longer possible and it is hard to get sufficient tissues for molecular testing (Luo et al. 2014). At least 8 randomized phase III studies have shown, in patients suffering from advanced NSCLC with EGFR mutation, superiority in patients on oral treatment with an EGFR tyrosine kinase inhibitor such as gefitinib (250 mg/day), erlotinib (150 mg/day) or afatinib (40 mg/day) in the first line of treatment compared to standard platinum-based chemotherapy, in terms of both response rate (RR) and progression-free survival (PFS) (Han et al. 2012; Mitsudomi et al. 2010; Maemondo et al. 2010; Zhou et al. 2011; Goto et al. 2012). Osimertinib is a third-generation EGFR-TKI that targets specifically the T790M-mutated cells; T790M is a resistance mutation that is found in nearly 50% of patients under first-line TKI treatment after a year. Its superiority over platinum-based treatment in terms of PFS, overall response rate (OR) and duration of response (DOR) has been demonstrated in the AURA 3 trial (Mok et al. 2016). Several studies have been carried out to assess the cases in which the EGFR gene mutation was present both in the blood sample and in the tissue finding, but the diagnostic accuracy has always revealed very wide range variation. The sensitivity for the analysis of circulating free DNA (cfDNA) ranges from 17 (Kim et al. 2013) to 100% (Punnoose et al. 2012; He et al. 2009), while the specificity fluctuates between 71.4 (Kimura et al. 2006) and 100% (Goto et al. 2012). The great variability that characterizes the different studies can be explained using different cfDNA laboratory extraction and sequencing procedures. Also, the dimensions of the tissue sample influence the sensitivity and specificity data (Sun et al. 2015). Two large meta-analysis studies (Qiu et al. 2015; Luo et al. 2015) were conducted to compare cfDNA with tumor tissue in terms of diagnostic accuracy for EGFR mutations. Both of these studies found that cfDNA has a high diagnostic accuracy to identify EGFR mutations in NSCLC patients. Both in these studies, the sensitivity of cfDNA has been shown to be 67.4% and 62%, respectively, and the specificity of 93.5% and 95.9%, respectively (Sun et al. 2015). Considering these data, the cfDNA is an attractive approach with a high specificity/sensibility to investigate mutations of the EGFR for treatment purposes. As the clinical need to quickly identify the proper patients for the proper treatment especially in NSCLC is crucial, the aim of the present study was to provide the importance of clinical use in a daily practice of liquid biopsy (LB) in NSCLC treatment.

## Materials and methods

### Patients

This is a retrospective, monocentric study, conducted on 30 patients with NSCLC treated at the Oncology Unit of Azienda Sanitaria Universitaria Integrata (ASUI) of Trieste (Italy), in the period between December 2016 and May 2019. Last follow-up update was on 21 May 2019. Patients with NSCLC, all of whom tested positive for mutational analysis of the EGFR gene at histological/cytological first evaluation (baseline), with signed informed consent, were included in the analysis.

### Diagnosis and molecular test of EGFR status

To perform the mutational comparison between the basal cell/tissue and the cfDNA analysis performed on liquid biopsy, the blood sampling (approximately 10 ml in EDTA tubes) has been taken at the same time of diagnosis or before starting the target treatment (T0). Patients who underwent surgical removal of tumor (Mitsudomi et al. 2010) performed LB at time of progression as no adjuvant treatment nor more mutational analyses were needed after surgery.

A second blood sample (T1) was proposed for patients who had been on therapy for at least 4 months and/or, according to clinician opinion and/or unclear CT results, had shown early signs of clinical disease progression (*n* = 19) to evaluate potential changes of the mutational status of EGFR during treatment. The LB has been performed at the same time of the CT scan to correlate the EGFR status with the disease with response/resistance to treatment.

Extraction, purification and concentration of circulating free DNA (cfDNA) were performed from 1 to 5 ml of plasma with “Helix Circulating Nucleic Acid kit” (Diatech Pharmacogenetics) within 60 min from blood withdrawal. The detection analysis has been performed with 100 μl by real-time PCR using the “Easy EGFR kit” or the “EasyPGX^®^ ready EGFR” kit (Diatech Pharmacogenetics). The kit allows the detection of the principal mutations, deletions or insertion on exons 18, 19, 20 and 21 of EGFR gene with a sensitivity of up to 0.5%.

### Statistical analysis

The clinical–pathological characteristics of the study population were described by means of ± standard deviation (SD) or median and range (minimum–maximum) for continuous variables, while with absolute and percentage frequencies for categorical variables. The association between presence or absence of mutation and the categorical variables of interest (type of mutation, type of withdrawal and clinical response of the patient) was evaluated by Fisher’s exact test. The proportions test was used to compare the difference in the percentage of mutated patients at baseline and the first plasma sample. The comparison of the mutational status between T0 and T1 was performed with the Mc-Nemar test for paired data and the degree of agreement was evaluated by Kappa index of Cohen (Landis and Koch 1977). For the patients in oncological therapy with targeted drug and with T1 sample available (*n* = 18), progression-free survival (PFS) and overall survival (OS) were evaluated: PFS was defined as the time from the beginning of the therapy to the date of the first progression and OS was defined as time between end of therapy and time of death or date of last visit for living patients. PFS and OS were estimated using the Kaplan–Meier method and the differences between the groups were tested using the Log-rank test. OS in % and PFS in % express, respectively, the percentage of patients alive and the percentage of patients free of disease at a specific time point. All statistical analyses were performed using the statistical software R (the R Foundation for Statistical Computing; Version 3.5.0) and the software STATA 14.2 (StataCorp, College Station, TX). *P* value values less than 0.05 were considered statistically significant.

## Results

All 30 patients included in the study had been diagnosed with NSCLC, stages I–IV, the vast majority of them being diagnosed already as metastatic. EGFR target therapy prescription is limited to advanced stages of NSCLC; therefore, all the patients included in the liquid biopsy analyses had either progressed or already had a stage IIIA–IV disease; clinical characteristics are reported in Table 1.

**Table 1:** Baseline characteristics of NSCLC patients

	*N*	%
Gender		
Female	23	76.6
Male	7	23.3
Age		
≥ 60 years	25	83.3
< 60 years	5	16.7
Age, years (mean ± SD)	69.58 ± 10.63	
Smoke		
Smoker	3	10.0
Non-smoker	19	63.3
Ex-smoker	4	13.3
NA	4	13.3
Stage of disease at diagnosis		
IA2	1	3.3
IB	1	3.3
IIB	1	3.3
IIIA	1	3.3
IIIB	2	6.6
IIIC	1	3.3
IV	20	66.6
NA	3	10.0
Histopathology		
Adenocarcinoma	30	100

*SD*  standard deviation, *NA*  not available

Baseline EGFR mutational analyses were performed using cytological samples in 7 patients (23.3%), on tissue biopsy in 16 patients (53.3%) and on sample after surgery on 7 patients (23.3%).

In 18 out of 30 patients (60%), the mutation detected at baseline was also confirmed on the cfDNA; while in 12 patients (40%), the mutation at baseline was not detected on the corresponding cfDNA (Table 2). The sensitivity of mutation status detection between baseline tumor and T0 samples for patients evaluable for both samples was 60.0% (95% CI: 41.0–77.0%).

**Table 2:** EGFR mutations status for tissue samples and T0

EGFR mutation status	Tissue samples *N* (%)	T0 *N* (%)
Deletion 19	14 (46.7%)	12 (40.0%)
Exon 21 (L858R)	14 (46.7%)	5 (16.7%)
Addition Exon 20	2 (6.7%)	1 (3.3%)
Mutation Negative	0 (0.0%)	12 (40.0%)
Total	30	30

**Fig. 1:**
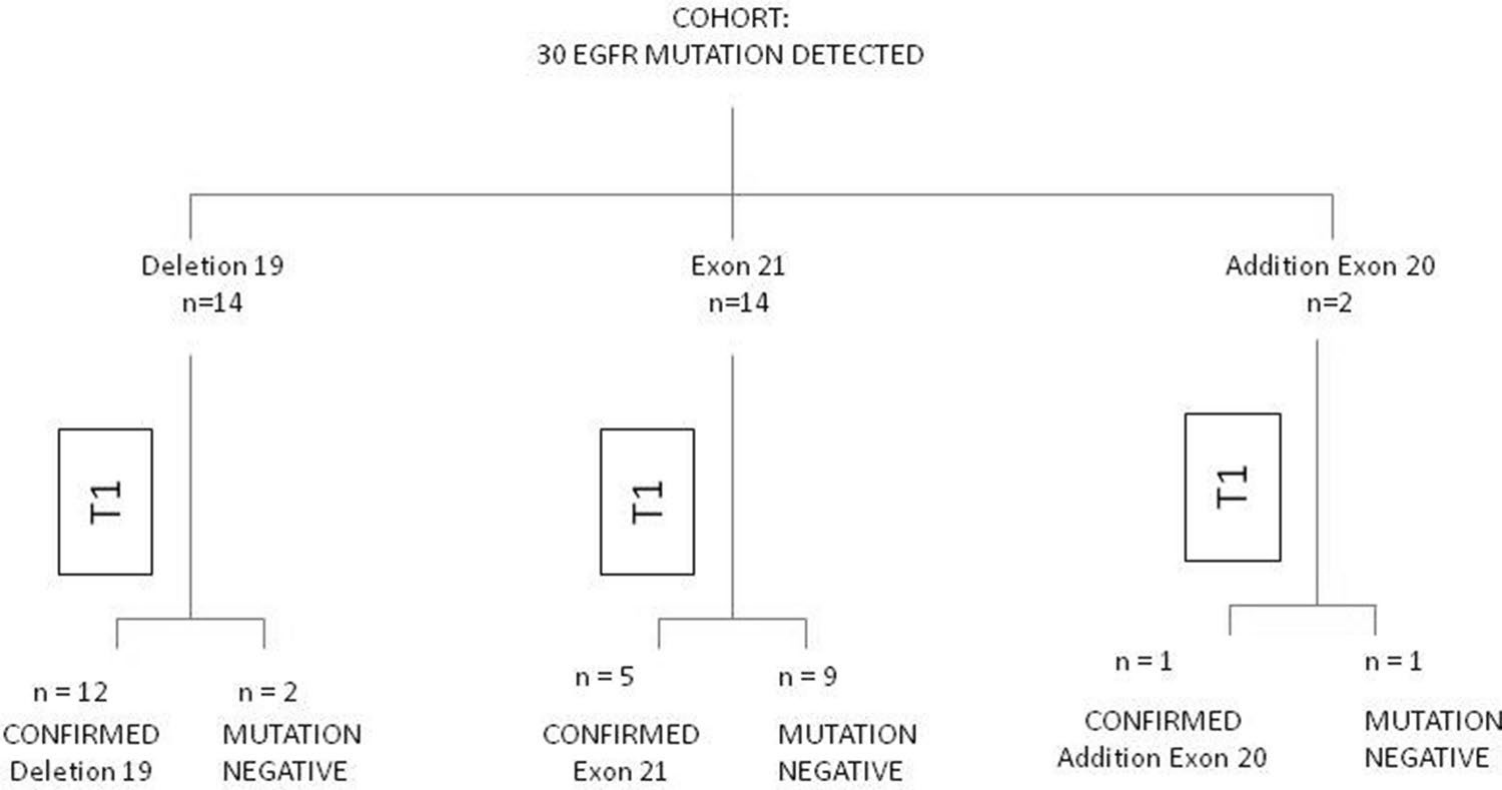
EGFR mutation status summary for tissue sample versus T0 circulating free DNA (patients evaluable for both samples, *n* = 30)

Comparing the analysis performed on cell/tissue versus cfDNA, we noted the absence of mutation is associated with the type of mutation itself (see Fig. 1). Discordance between the absence of detection of the mutation was greater in patients carrying the exon 21 mutation (9 out of 14 patients, 64.3%) than with the deletion of exon 19 (2 out of 14 patients, 14.3%) or mutation in exon 20 (1 of 2 patients, 50.0%) (*p* = 0.02, Fisher Exact test).

Of the 7 patients with baseline mutational analysis on the surgical sample, 3 had stage IV disease, and one each had stage IB, IA2, IIIA, one patient had no available information on stage. Stage at diagnosis for the 23 patients with baseline mutational analyses on biopsy (histological or cytological) was not available for 2 patients, one patient had IIB and one IIIC stage, 2 patients had IIIB and 17 patients had stage IV disease.

Patients with a mutation detected at baseline with FNA or biopsy showed the same mutation detected on cfDNA (*n* = 5, 71.4% and *n* = 11, 68.8%, respectively); On the other hand only 2 out of 7 patients with a surgical mutated tissue confirmed the mutation on cfDNA (28,6%). Thus, overall, a trend association is observed between samples (FNA and biopsy versus surgery) on which the mutation at baseline was performed and the mutational state at T0 (*p* = 0.08, Fisher Exact test).

Moreover, a mutational analysis on cfDNA was performed on 19 patients (63.3%) during treatment with anti-EGFR-TKI. In 9 out 19 patients, the same mutation was detected in T0 and in T1; while in 4 patients, we found a change from mutation to no mutation detected during the therapy. No change from no mutation to mutation has been detected during the two time points and in 6 out 19 patients, no mutation has been detected, neither in T0 nor in T1.

All of the patients that had a confirmed mutation on T1 (9, 100%) are in a progression state (*p* = 0.003, Fisher Exact Test). The percentage decreases both for patients not mutated on both samples (2, 33.3%) and for mutation-negative patients (1, 25%) (Table 3).

**Table 3:** Distribution of patients respect to concordance/discordance between mutational status detection at T0 and T1 and between therapy response evaluation

Mutational status on T0 and T1	*n*	PD	SD	12-month PFS (95% CI)	12-month OS (95% CI)
Mutation at T0–mutation at T1	9	9	0	22.2% (6.5–75.4%)	55.6 (20.4–96.1%)
Mutation at T0–no mutation at T1	4	1	3	50.0% (18.8–100%)	75.0% (12.8–96.1%)
No mutation at T0–no mutation at T1	6*	2	4	66.7% (27.2–100%)	100% (1.00–1.00)

*PD* progressive disease, *SD* stable disease, *PFS* progression-free survival, *OS* Overall survival

*5 are included in the survival analysis: one patient excluded because of progressive disease prior to EGFR-TKI treatment and received first-line therapy

**Fig. 2:**
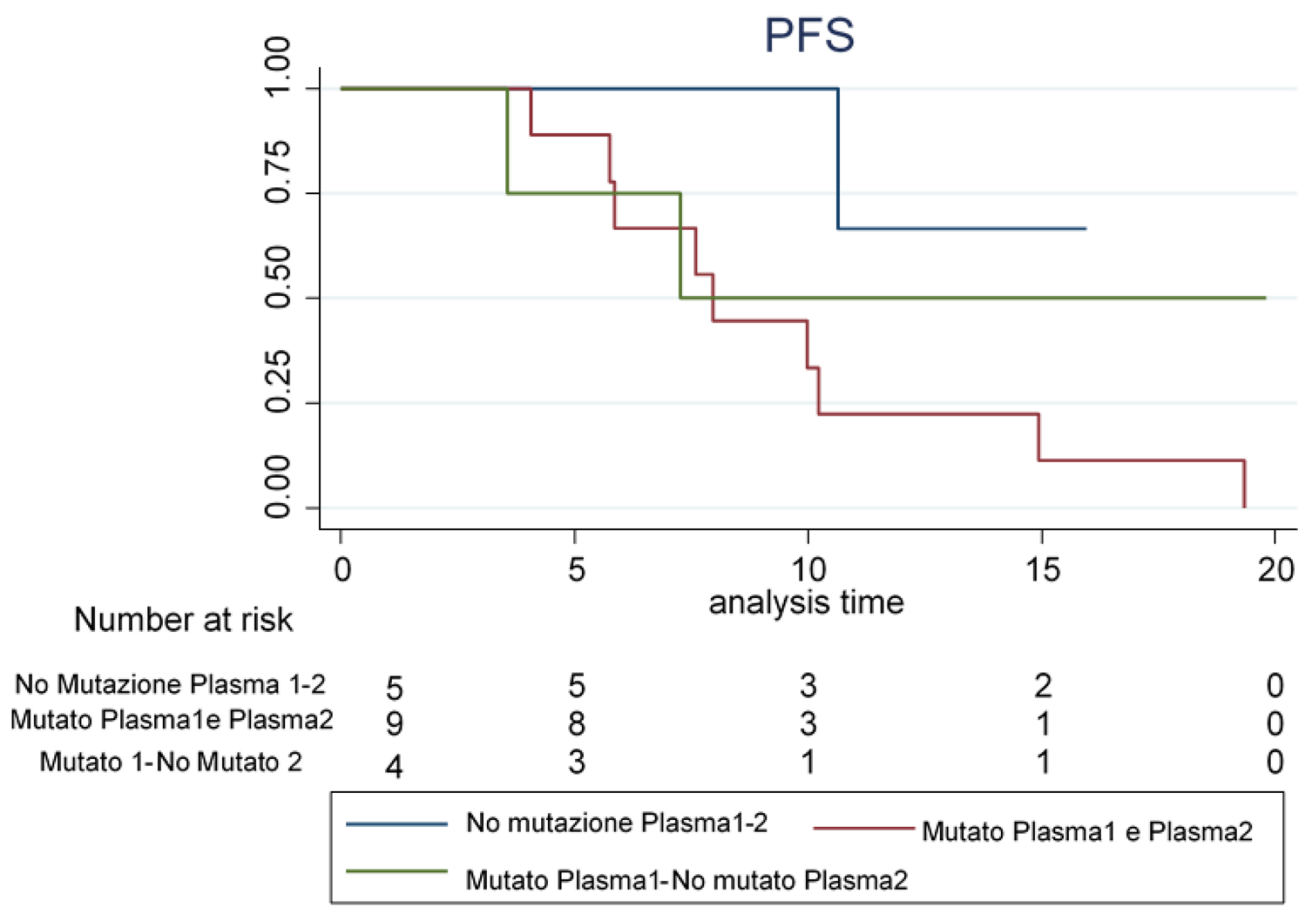
Kaplan–Meier progression-free survival (PFS) curves for patients treated with target oncological therapy with respect to EGFR mutational status detected in T0 and T1 samples

In the survival analysis were only included patients who received EGFR-TKI therapy (22, 73.3%): 10 patients received gefitinib, 8 afatinib and 4 erlotinib. Moreover, two of the 22 patients had progressive disease prior to EGFR-TKI treatment and received first-line therapy; other two patients did not have second blood withdrawal available, and were, therefore, excluded from survival analysis. We compared the Progression-Free Survival (PFS) curves between three groups: no mutation detected at both T0 and T1 (*n* = 5, median PFS has not yet been reached—more than half of patients were still living), mutation on both plasma samples (*n* = 9, median PFS = 7.97 months), mutated on T0 but with no mutation detected on T1 (*n* = 4, median PFS = 7.27 months) (Fig. 2). Patients without mutation detection at both time points showed the longest PFS at 12 months (66.7%, 95% CI: 27.2–100%); whereas, patients who maintained a mutation during treatment showed the shorter PFS (22.2%, 95% CI: 6.5–75.4%). Patients with a discordance in the mutational status detected on cfDNA on the two time points showed an intermediate situation in terms of PFS at 12 months (50.0%; 18.8–100%).

**Fig. 3:**
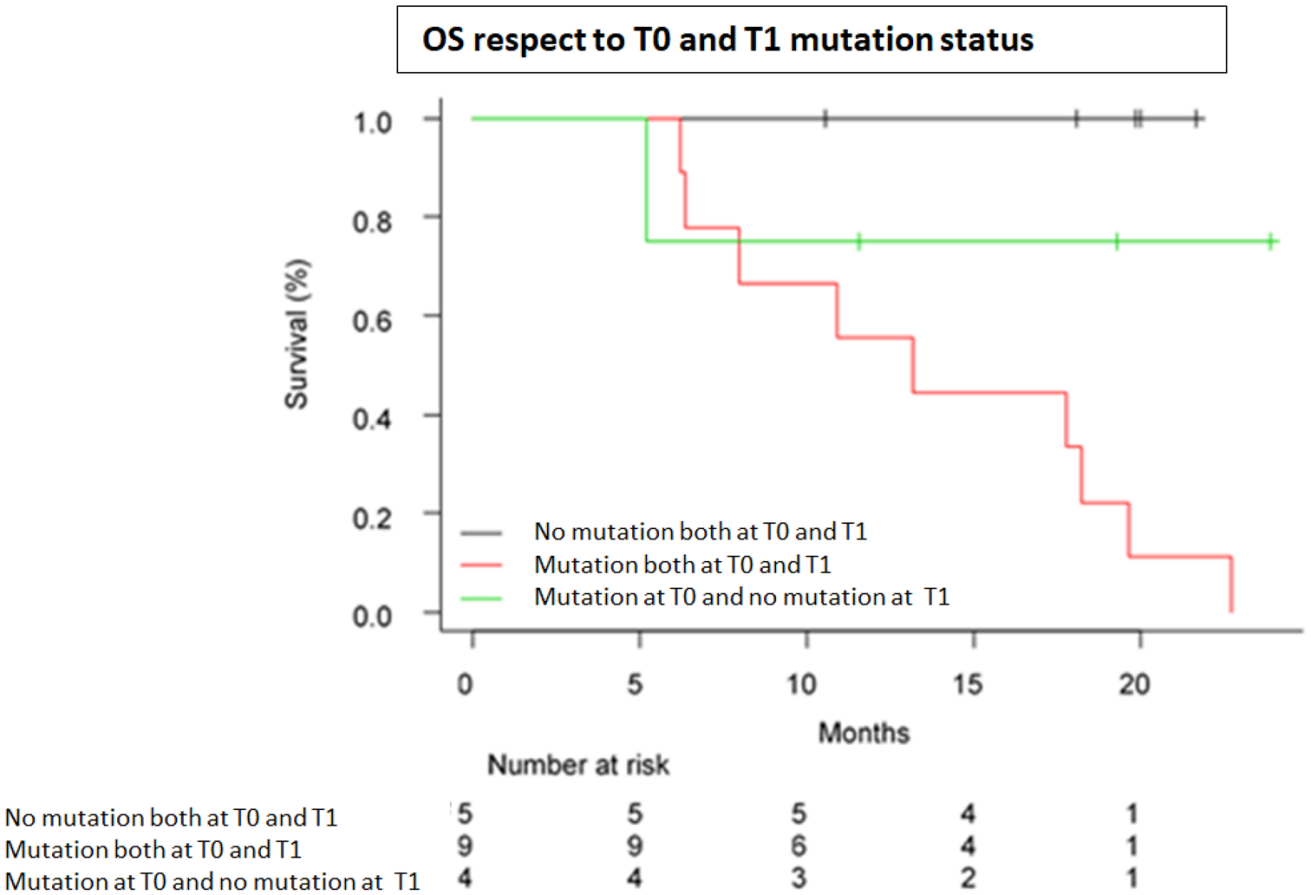
Kaplan–Meier overall survival (OS) curves for patients treated with target therapy with respect to EGFR mutational status detected at T0 and T1

The median OS at 12 months was 18.21 months (95% C.I.: 7.97-NA) with 12 events occurring as reported in other studies on EGFR-mutated NSCLC patients treated with target therapy (Douillard et al. 2014). Patients whose basal mutation was confirmed by liquid biopsy at T0 (*n* = 13), had a 12 months OS of 53.8% (95% CI: 26.7–74.7%) with a median OS of 13.2 months. On the other hand, all of the patients with a discordance between cyto/histological and plasma mutation (EGFR mutation not detected on T0) were alive at the 12th month of follow-up (*n* = 5; OS 100%; 95% CI: 100–100) (*p* = 0.02, Log-Rank test). The overall survival at 12 months was then evaluated between basal and T1 mutation. The OS was 88.9% (95% CI: 43.3–98.4%) for patients (*n* = 9) who did not reveal the mutation at T1 and decrease significantly to 55.6% (95% CI: 20.4–80.5%) in patients (*n* = 9) mutated both at basal and T1 (*p* = 0.004). Lastly, the OS was evaluated in relation to mutation status between T0 and T1 (Fig. 3). The 5 patients who resulted negative both at T0 and T1 were all alive at 12 months (OS = 100%, 95% CI: 100–100); 4 patients who had a positive T0 but negative T1 registered an intermediate OS (75.0%, 95% CI: 12.8–96.1%) and 9 patients who tested positive both at T0 and T1 had a 12-month OS of 55.6% (95% CI: 20.4–96.1%). Differences between groups were statistically significant (*p* = 0.01). Table 3 clarifies the distribution of patients based on concordance/discordance between mutational status detection at T0 and T1, therapy response evaluation and 12-month PFS and OS.

## Discussion

Onset of drug resistance in NSCLC EGFR-mutated patients undergoing first-line tyrosine kinase inhibitors treatment usually occurs between 9 and 12 months of therapy (Chen et al. 2019), which leads to the need to follow as closely as possible the evolving of the disease. The distribution of the mutations’ frequency and the sensitivity, understood as the detection rate of the liquid biopsy for the mutational structure, were comparable to literature data (0.61; 0.50–0.71, 95% CI) (Qiu et al. 2015; Luo et al. 2015). Real-time PCR, despite being often seen as an outdated technique, guarantees reproducibility and applicable and expendable standards in clinical practice. On the other hand, it limits the analysis to the region of interest of the EGFR gene and does not allow detection of rare mutations. There was a reduced relationship between the mutation present at baseline on the surgically excised tissue and that identified in the plasma. A hypothesis that could possibly explain this difference lies in the therapeutic choice, as the surgery itself. The absence of mutation at the cfDNA plasma indicates that the surgery has effectively eradicated the tumor or that the number of tumor cells, responsible for releasing the tumor DNA into the circulation, has decreased to the point that they are undetectable. Furthermore, patients not eligible for surgery have generally higher stage disease (metastatic stage in 18 out of 23 patients); this is an indication of a more aggressive disease and higher tumor burden that can justify the higher concordance between basal and liquid mutation detection in respect to analysis performed on surgical samples.

All patients for whom EGFR mutation was identified in plasma assessment both at T0 and T1 are in a state of disease progression (PD) (9, 100%), while the percentage of progressive disease falls both for patients who preserved a no-mutation-detected profile (2, 33.3% of PD) and for those who had a first mutated profile but changed to no mutation detected in the second sample (1, 25%).

The evaluation of the second plasma sample allows the evaluation of the response to treatment and the possible insurgence of any mutations responsible for resistance to therapy. In 2 patients, it was identified the positivity to the T790M mutation of exon 20, a factor involved in the mechanisms of resistance to anti-EGFR generation I and II drugs and that makes the patient eligible for treatment with an anti-EGFR-TKI of third generation, osimertinib.

In terms of PFS, patients who changed their mutational profile (on LB) from mutated to no mutation detected had a worse prognosis than the patients whose mutation is not detected in T0 and 2 but a better prognosis in respect to those who maintained the mutation over time. Due to the small samples size of the analyzed cohort, the results were not statistically significant; however, patients with both T0 and T1 EGFR mutations showed a trend for worse progression-free survival compared to patients without EGFR mutations detected on ctDNA (*p* = 0.14, Log-Rank Test). Similarly, the detection of a plasma mutation held a negative prognostic value in terms of OS. Indeed patients whose mutation is detected at both plasma timepoints registered the lowest OS (55.6%, 95% CI: 20.4–96.1%). All of the patients who maintained a status of no mutation detection on liquid biopsy were alive at time of analysis. This preliminary study showed that the “non-detection” of EGFR mutation by LB during target treatment fosters a longer survival, and that the earlier the negativization of the mutation detection, the better the prognosis.

Therefore, even with the limitation due to the small number of patients included in this preliminary analysis, the negativization of the EGFR mutation evaluated by liquid biopsy at a second plasma sampling seems to coincide with a prognostic improvement.

## Conclusion

The results obtained and the high concordance between cyto/histological and liquid evaluation (60%, 95% CI: 41.0–77.0%) described in this study allow us to state that liquid biopsy (LB) today is a valid technique to use in clinical routine. At the present time, international guidelines allow prescription of first-line EGFR-TKI therapy based only on LB in those patients for whom a surgical procedure or biopsy is infeasible, or the amount of tumor tissue is scarce (Rolfo et al. 2018).

The status and modification of EGFR mutations and the occurrence of new mutations related to drug resistance during therapy with anti-EGFR-TKI drugs determined on cfDNA showed a correlation with PFS and OS. Indeed, the negativization of the plasma mutation detection was consistent with a longer PFS and so with a better prognosis. The main limitation of this study is the low number of patients. If these data would be confirmed on a larger scale, monitoring EGFR mutation via liquid biopsy in NSCLC patients, particularly in advanced stages, could give the clinician a prediction on treatment response, disease progression and survival itself, fostering a modified scheduled monitoring based on plasma mutational status. Therefore, we endorse the use of cfDNA EGFR liquid biopsy as a validated instrument of clinical significance.

## Data Availability

Data are available from the corresponding author on reasonable request.
